# Supplementary material for: Thalamocortical feedback selectively controls pyramidal neuron excitability
Source: Nat Commun. 2025 Jul 1;16:5663. doi: 10.1038/s41467-025-60835-w (PMC12215560; doi:10.1038/s41467-025-60835-w)
Supplement: Supplementary file 1 — Supplementary Information [file 41467_2025_60835_MOESM1_ESM.pdf]

Supplementary Information for:

Thalamocortical feedback selectively controls pyramidal neuron excitability.

Federico Brandalise*[1,2], Ronan Chéreau*[1], I-Wen Chen[1], David van Oorschot[1], Claudia Morin Raig[1], Tanika Bawa[1], Nandkishor Mule[1], Stéphane Pagès[1,3], Foivos Markopoulos[1], Anthony Holtmaat[1#]

[1]Department of Basic Neurosciences and the Center for Neuroscience, Centre Médical Universitaire (CMU), University of Geneva, 1211 Geneva, Switzerland

[2]*Current address:* Department of Biomedical Sciences, Div. Neuroscience and Clinical Pharmacology, University of Cagliari, Italy

[3]*WYSS center, Campus Biotech, Geneva*

*These authors contributed equally to this work

[#]Corresponding author's email: anthony.holtmaat@unige.ch

Supplementary Information

Supplementary Figures 1-17

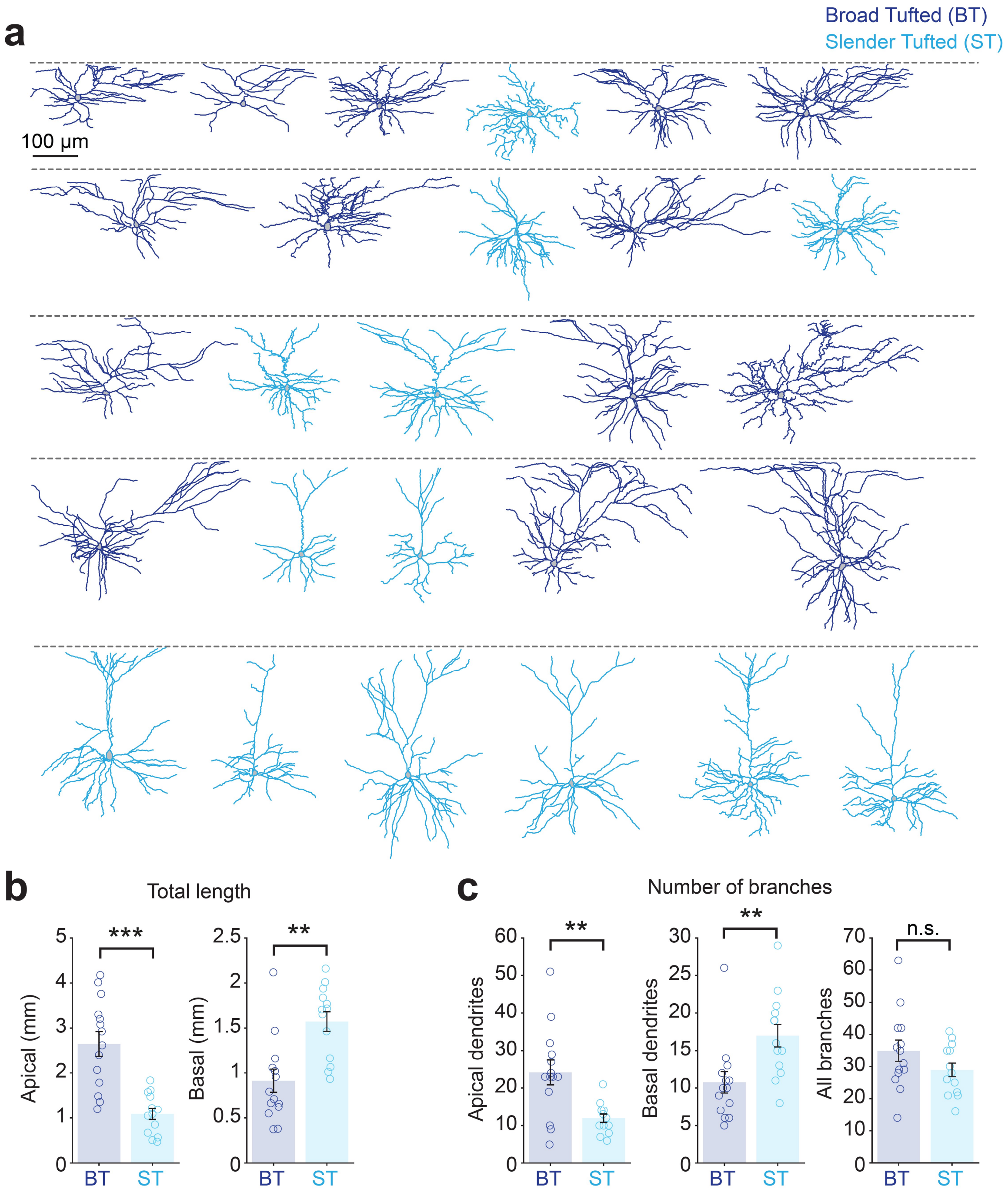

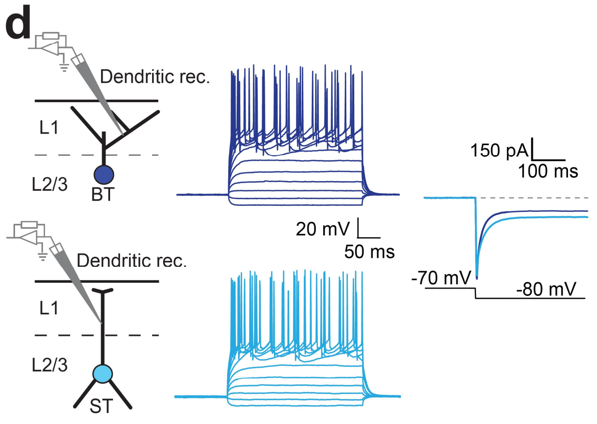

e

**Passive properties**	BT (n = 30)	ST (n = 34)	P value
V_m (mV)	-73.20 ± 3.75	-72.4 ± 2.90	0.87
R_{in} (mΩ)	161.0 ± 16.0	177.4 ± 18.0	0.38
τ_{fast} (ms)	0.43 ± 0.06	0.68 ± 0.13	0.08
τ_{slow} (ms)	2.2 ± 0.2	4.2 ± 0.8	< 0.02 *
Capacitance (pF)	43.1 ± 2.56	78.4 ± 4.7	< 0.01 **

Supplementary Fig. 1 | L2/3 pyramidal neurons in the S1 can be segregated into two groups based on their morphology and electrophysiological features (related to Fig. 1).

a Morphological reconstructions of the dendritic arborization of 27 recorded and biocytin-filled L2/3 pyramidal neurons. Neurons are sorted ascendingly by the depth of their soma relative to the pia (represented by a dotted line). BT and ST neurons were segregated based on the clustering analysis of the dendritic span and density within the first 200 µm (cf. Fig. 1, BT neurons are colored in dark blue and ST neuron are colored in light blue). **b** BT neurons have longer apical dendrites than ST neurons ($n = 14$ BT and 13 ST neurons; $P = 1.7\times10^{-4}$, Wilcoxon rank-sum test) but have shorter basal dendrites ($P = 0.0015$, Wilcoxon rank-sum test). **c** BT neurons have more apical branches as compared to ST neurons ($n = 14$ BT and 13 ST neurons; $P = 0.005$, Wilcoxon rank-sum test) but have fewer basal dendrites ($P = 0.003$, Wilcoxon rank-sum test). On the right, the comparison of the total number of branches is not significantly different between BT and ST neurons ($P = 0.17$, Wilcoxon rank-sum test). **d** Example dendritic recording traces of a BT (top) and a ST neuron (middle) in response to a series of current steps (starting at -150 pA and increasing in 100 pA increments) displaying very similar firing properties (in these examples, both cells have a rheobase of 450 pA). Note that the action potential thresholds are somewhat higher than what is typically observed in somatic recordings (somatic rheobase: 190 ± 49 pA, $n = 5$; dendritic rheobase: 410 ± 110 pA, $n = 12$), which is likely due to the large electrotonic distance of the dendritic recording site to the AP initiation site. The passive properties were assessed by analyzing the changes in current to a hyperpolarizing step from -70 mV to -80 mV (right). **e** Summary table of the passive properties of BT and ST neurons (Wilcoxon signed-rank test used for all parameters). Data displayed as mean ± s.e.m. Source data are provided as a Source Data file.

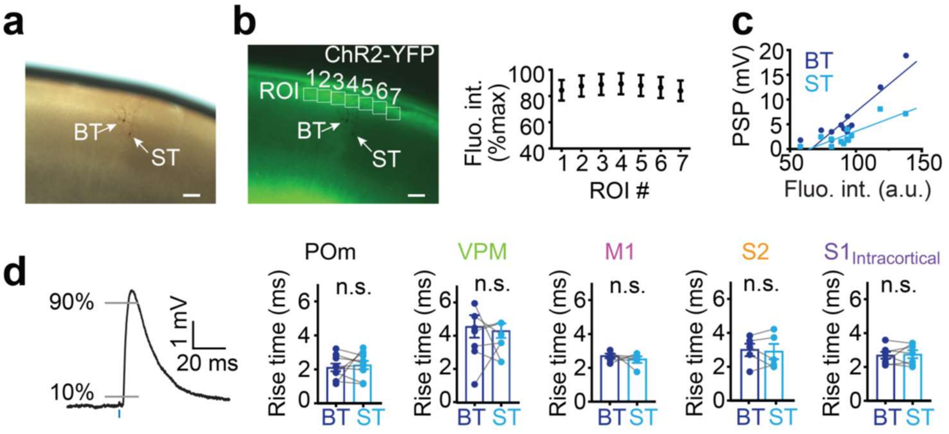

Supplementary Fig. 2 | Data pairing of BT and ST neurons within brain slices to account for the variability of opsin expression of the various inputs (related to Fig. 2).

a Example of an acute brain slice where a BT and a ST neuron were recorded and biocytin-filled. To compare the relative weight of the various inputs to BT and ST neurons by accounting for the variability of the opsin expression, dendritic recordings were performed on both cell types in each brain slices. **b** YFP signal reflecting the ChR2 expression in thalamocortical projections from the POm in the same brain slice as shown in A (left). To assess the homogeneity of the opsin expression in L1, a series of regions of interest (squares of 50 µm) were drawn below the pia, in which the average fluorescence of the YFP signal was measured. In this example, the quantification shows that for these neighboring recorded cells, the expression is relatively similar (right). Scale bars are 100 µm. **c** PSP response amplitudes from ST and BT dendritic recordings, evoked by POm photostimulation as a function of the average YFP expression. This reveals a linear correlation (for BT neurons, $R^2 = 0.83$; for ST neurons, $R^2 = 0.73$, $n = 11$ pairs of neurons and slices), indicating that stimulations were performed under non-saturating conditions. **d** To verify that the differences in amplitude observed between BT and ST neurons were not explained by the dampening of the PSPs due to different electronic distances, PSP rise times were compared for all measured inputs. The rise time of a PSP was defined as the time difference between the 10% to the 90% of the maximum value of the PSP. No difference in rise time between BT and ST neurons was observed for all the tested inputs (for POm, BT: 2.1 ± 0.2 ms, $n = 9$, ST: 2.3 ± 0.3 ms, $n = 9$, $P = 0.64$; for VPM, BT: 3.9 ± 0.6 ms, $n = 7$, ST: 3.7 ± 0.4 ms, $n = 7$, $P = 0.75$; for M1, BT: 2.7 ± 0.1 ms, $n = 5$, ST: 2.5 ± 0.2 ms, $n = 5$, $P = 0.58$; for S2, BT: 3.0 ± 0.4 ms, $n = 5$, ST: 2.9 ± 0.4 ms, $n = 5$, $P = 1$; for $S1_{intracortical}$, BT: 2.6 ± 0.2 ms, $n = 7$, ST: 2.7 ± 0.2 ms, $n = 7$, $P = 0.78$, Wilcoxon signed-rank tests). Source data are provided as a Source Data file.

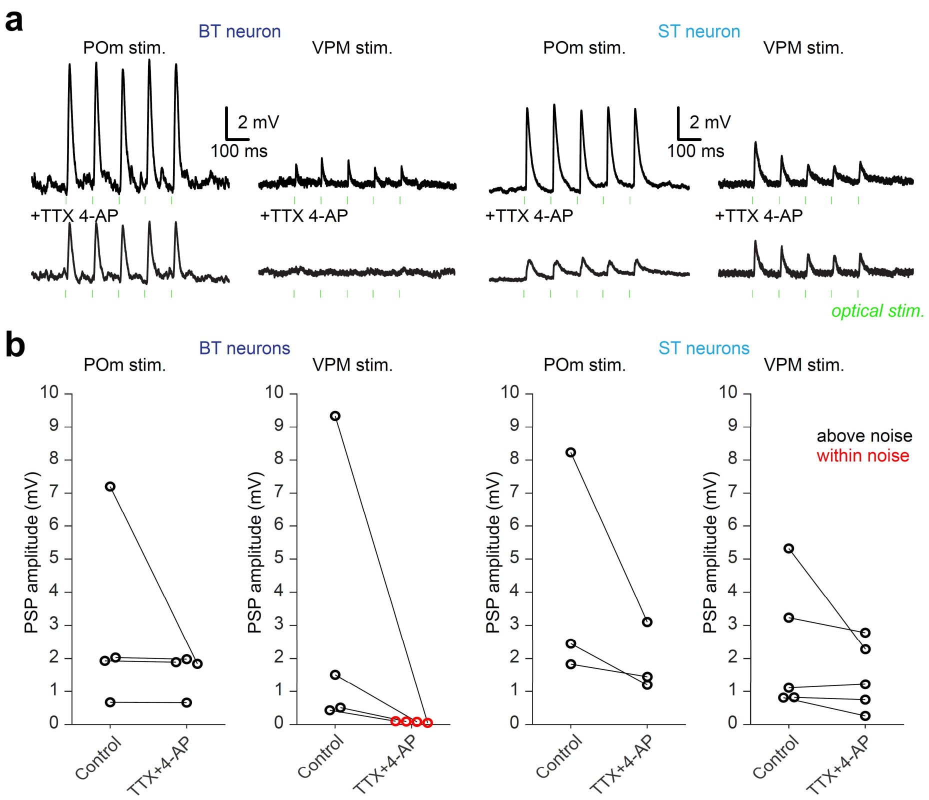

Supplementary Fig. 3 | Comparison of POm and VPM monosynaptic input to BT and ST neurons (related to Fig. 2).

a Dendritic recording in a BT neuron (left) and a ST neuron (right) showing the evoked PSPs when opsin-expressing POm and VPM afferents are photostimulated independently (5 pulses of 1 ms at 8 Hz) before and after bath application of TTX (1 µM) and 4-AP (100 µM) to reveal the direct monosynaptic response. The responses to the POm stimulations were reduced for both neuron types. However, the responses to the VPM photostimulations were completely abolished in the BT neuron. **b** Comparison of the mean amplitude of the PSPs upon POm or VPM stimulations for BT and ST neurons in control versus TTX + 4-AP conditions. Notably, the application of TTX + 4-AP fully abolished the response to VPM stimulation in BT neurons. The noise level of the recording was determined as the minimum and maximum values of the pre-stimulation baseline period (for BT neurons, POm stimulation, $n = 4$, VPM stimulation, $n = 4$; for ST neurons, POm stimulation, $n = 3$, VPM stimulation, $n = 5$). Source data are provided as a Source Data file.

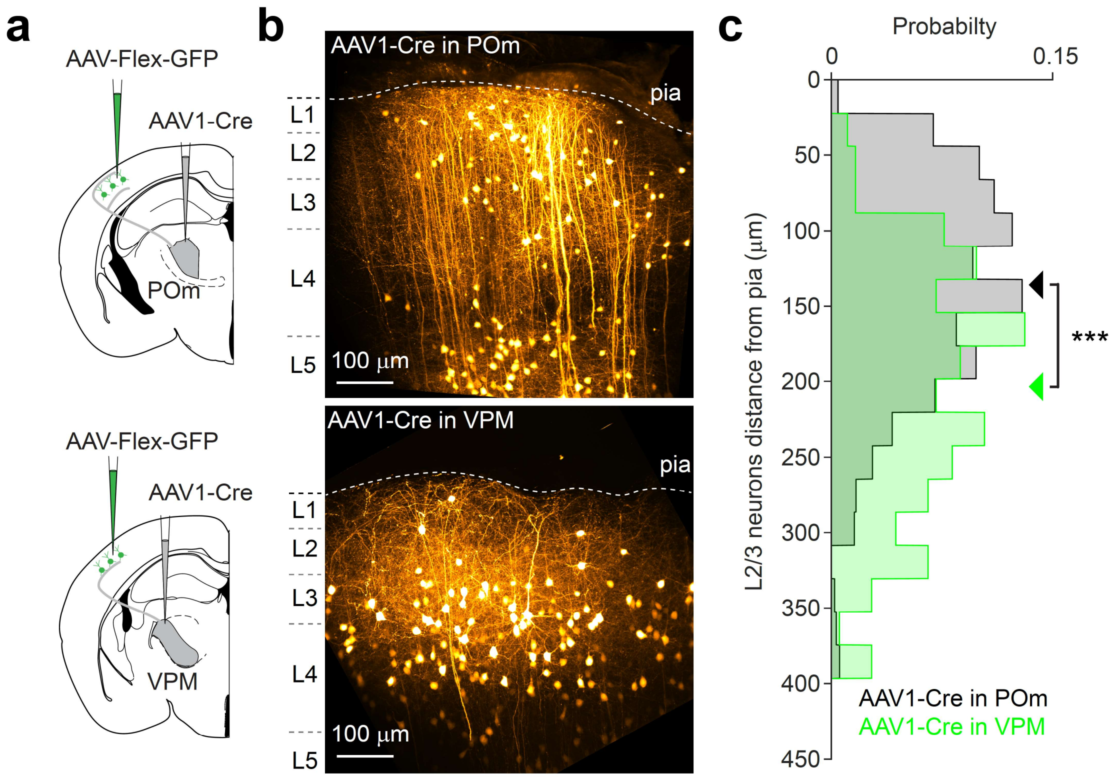

Supplementary Fig. 4 | Trans-synaptic AAV delivery from POm and VPM to S1 neurons (related to Fig. 1 and 2).

a An AAV1 vector expressing Cre recombinase was injected locally into either the POm or VPM. This allowed for anterograde trans-synaptic expression of Cre recombinase in postsynaptic neurons, including those in the S1 barrel cortex. Subsequently, a second AAV vector expressing a Cre-dependent conditional GFP reporter was injected into the S1 barrel cortex, enabling selective expression of GFP in the subset of neurons that received input from the targeted thalamic neurons and therefore expressed Cre recombinase. **b** Standard deviation projections of 2-photon image stacks of the GFP-expressing neurons in S1 when AAV1-Cre was injected in the POm (top) and in the VPM (bottom). Scale bars are 100 µm. **c**, Distribution of L2/3 neurons' somata depth expressing GFP. The anterograde trans-synaptic labeling from the POm targeted more superficial L2/3 neurons than the VPM (For POm: mean ± s.d., 136 ± 70 µm, n = 897 neurons from 5 mice; For VPM: mean ± s.d., 204 ± 79 µm, n = 183 neurons from 2 mice, $P = 4.52\times10^{-17}$, Kolmogorov-Smirnov test). Source data are provided as a Source Data file.

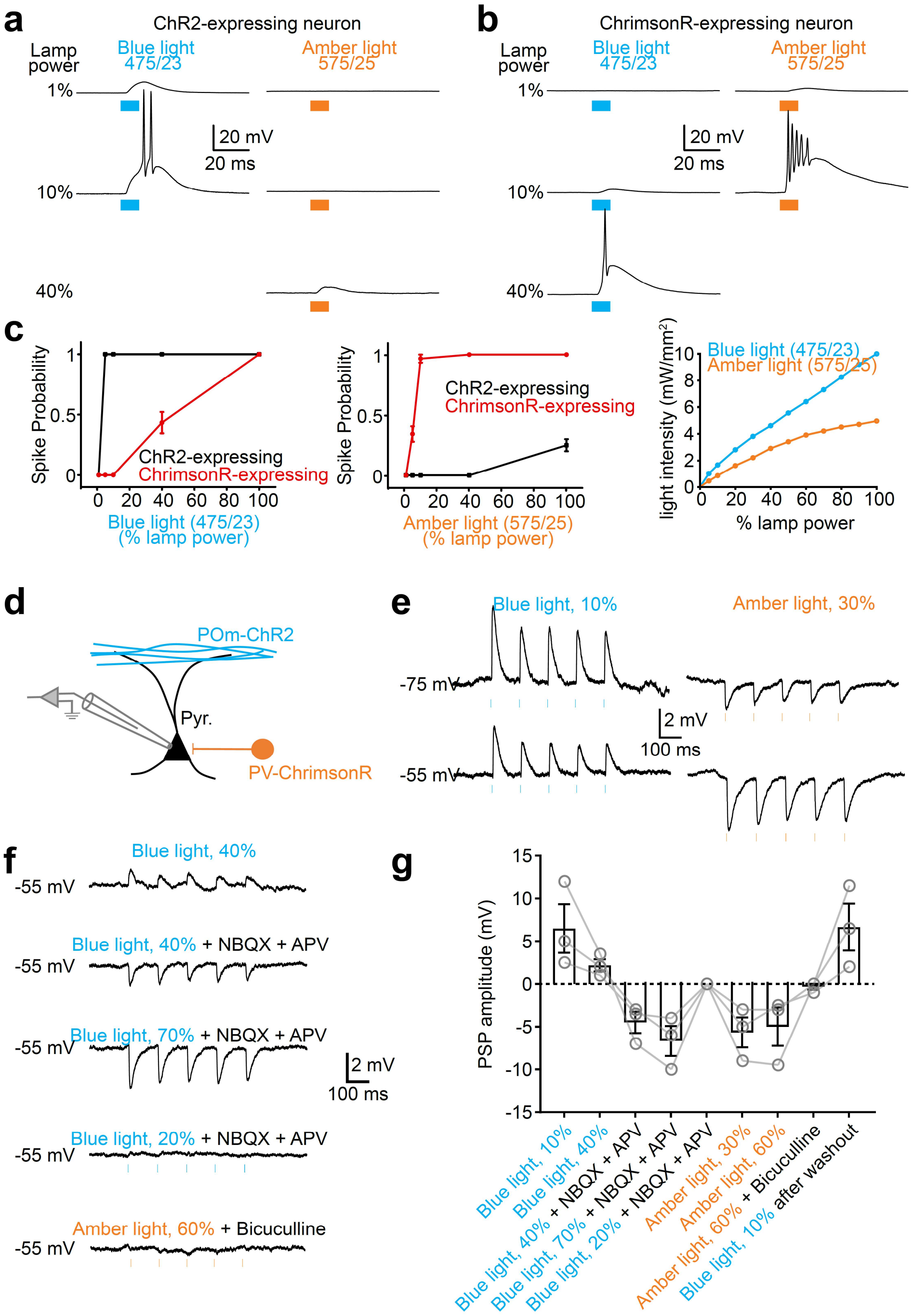

Supplementary Fig. 5 | Calibration of the light dose in the double-opsin experiments (related to Fig. 3).

a Somatic patch-clamp recordings and photostimulation of a ChR2-expressing neuron with a 10-ms pulse of blue light (475/23 nm) or amber light (575/25 nm) at various intensities. The neuron spiked when 10% of the blue light power is used whereas subthreshold activity is evoked with 40% of amber light power. **b** Same experiment with a neuron expressing ChrimsonR. In this case, 10% of amber light is sufficient to elicit spiking but strong blue light power (40%) may induce spiking as well. **c** Left, spike probability as a function of blue light power for ChR2- and ChrimsonR-expressing neurons (black and red lines, respectively). Using blue light power at 10% reliably made the ChR2-expressing neurons spike with minimal activation of the ChrimsonR-expressing neurons. Center, spike probability as a function of

amber light power for ChR2- and ChrimsonR- expressing neurons. Again, a lamp power of 20% maximized the spike probability of ChrimsonR-expressing neurons without exciting ChR2-expressing neurons ($n = 3$ neurons, probability calculated from 20 trials for each opsin and each power intensity). On the right, correspondence of the blue and amber light intensity as a function of the lamp power expressed in percentage, measured after the objective. **d** Test of the light-evoked synaptic responses in L2/3 pyramidal neurons from independent excitation of two different inputs. Specific expression of ChrimsonR in PV interneurons was performed by injecting a Cre-dependent AAV vector in S1 of a PV-Cre transgenic mouse, and a local injection of a non-Cre dependent AAV vector was used to express ChR2 in the POm. **e** Examples of current-clamp recordings of L2/3 pyramidal neuron responses to blue or amber light stimulation with a train of 5 pulses of 1 ms at 8 Hz. At resting membrane potential, 10% blue light elicits excitatory PSPs (EPSPs) while 30% amber light elicits inhibitory PSPs (IPSPs). Holding the membrane potential at -55 mV (as it was done for most of the experiments in this study) only affects the amplitudes of the responses due to a change in the driving force but not their sign. **f** Increasing the blue light power up to 40% decreases the EPSP amplitude due to the simultaneous activation of ChrimsonR in PVs. Blocking the glutamatergic response using 10 µM NBQX and 50 µm APV reveals the inhibitory component of the response, confirming the contamination of the response from ChrimsonR activation at this power. Increasing the blue light power up to 70% amplifies the IPSP response while 20% of blue light power does not elicit any IPSP. In another condition, increasing the power up to 60% when the GABAergic response is blocked with 10 µM bicuculline does elicit any EPSP. **g** Averaged PSP amplitude obtained with various light stimulations and bath applied drugs. The data indicates that ChrimsonR is not activated when the blue light power is below 20% and ChR2 is not activated when the amber light power is below 60% of the maximum lamp power ($n =$ 3 neurons). For the experiments in our study, we typically used 1.6 mW/mm^2 (10% of the maximum LED power) for blue light and 2.2 mW/mm^2 (30% of the maximum LED power) for the amber light, which is well below these values, and thus avoiding cross-contamination. Source data are provided as a Source Data file.

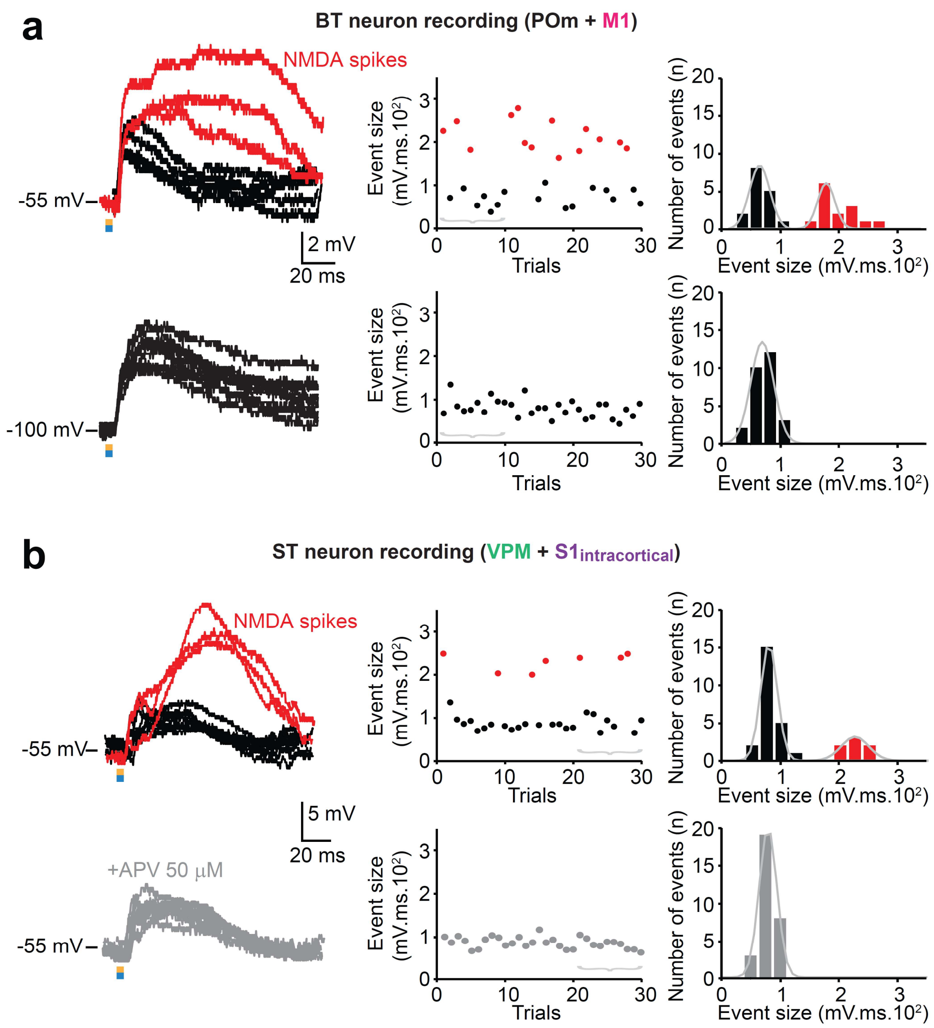

Supplementary Fig. 6 | NMDA spikes are prevented under hyperpolarized conditions and can also be evoked in ST neurons (related to Fig. 3).

a Dendritic recording of a BT neuron in S1 during the co-stimulation of POm and M1 inputs. NMDA spikes were observed when the cell was slightly depolarized at -55 mV (top) but were prevented when the cell was hyperpolarized at -100 mV (bottom). While NMDA spikes were prevented at -100 mV, the regular PSP event sizes did not differ from the -55 mV condition. **b** Dendritic recording of a ST neuron in S1 during the co-stimulation of VPM and $S1_{intracortical}$ inputs displaying NMDA spikes (top), which can be prevented by bath application of APV (50 µM; bottom). Brackets indicate the trials for the traces on the left. Source data are provided as a Source Data file.

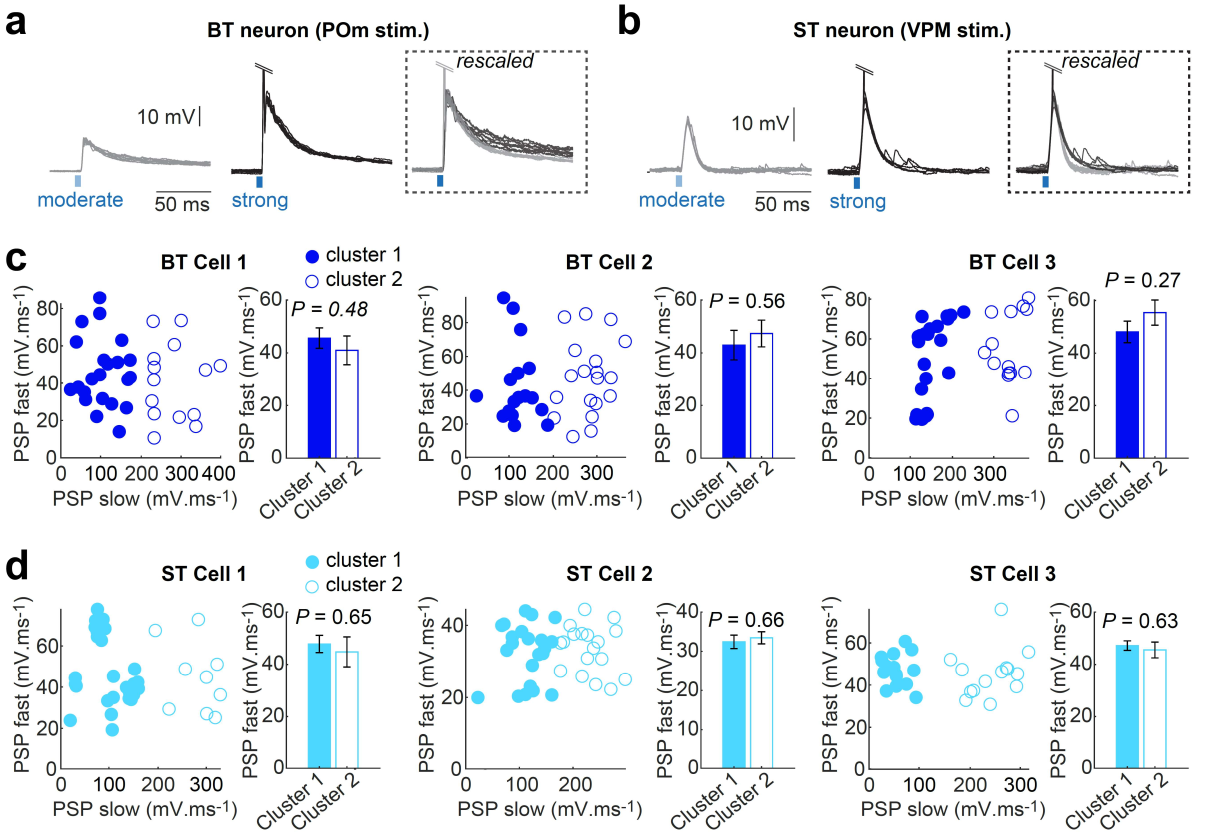

Supplementary Fig. 7 | The induction of NMDA spikes does not depend on the stimulation strength and the initial PSP size (related to Fig. 3).

a,b Dendritic recordings in a BT (**a**) and ST (**b**) neuron following moderate and strong photo-stimulation of POm (**a**) and VPM (**b**) afferents. Whereas the amplitude of the PSP increases upon strong stimulation, the shape remains similar (see inset) and no NMDA spikes were induced, even in trials where action potentials were elicited. **c** Relationship between the fast and slow components of PSPs in 3 example BT neurons that exhibited NMDA spikes upon the co-stimulation of POm and M1. A *k*-means clustering analysis, with $k = 2$, was used to separate events with NMDA spikes (open circles) from regular PSPs (closed circles). The bar graphs show that the average strengths of the fast PSP components were similar between the 2 clusters, indicating that the initial PSP size does not influence the generation of NMDA spikes. **d** Same analysis for 3 example ST neurons that exhibited NMDA spikes upon the co-stimulation of VPM and intracortical S1. Source data are provided as a Source Data file.

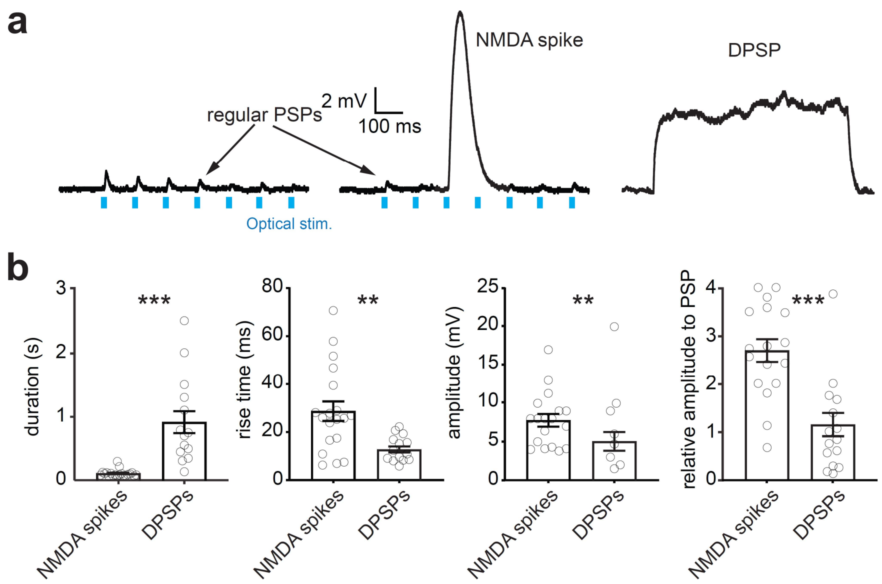

Supplementary Fig. 8 | Comparison between NMDA spikes and DSDPs (related to Fig. 4).

a Examples of dendritic recordings of a BT neuron during simultaneous photostimulation of POm and M1 afferent inputs. Evoked PSPs, NMDA spikes and delayed DSDPs were observed in this recording. **b** Comparison of the duration, rise time, amplitude and relative amplitude to PSP between NMDA spikes and DSDPs. DSDPs are characterized by a longer duration (for DSDPs, 912.9 ± 170.9 ms, $n = 16$; for NMDA spikes, 112.7 ± 12.3 ms, $n = 18$, $P < 0.001$, Mann-Whitney test), a faster rise-time (for DSDPs, 12.7 ± 1.2 ms, $n = 16$; for NMDA spikes, 28.7 ± 4.0 ms, $n = 18$, $P = 0.0018$, Mann-Whitney test), a smaller amplitude (for DSDPs, 5.0 ± 1.2 mV, $n = 16$; for NMDA spikes, 7.7 ± 0.8 mV, $n = 18$, $P = 0.0079$, Wilcoxon test), and a smaller relative amplitude to PSP (for DSDPs, 1.1 ± 0.2, $n = 16$; for NMDA spikes, 2.7 ± 0.2, $n = 18$, $P < 0.0001$, Mann-Whitney test). Source data are provided as a Source Data file.

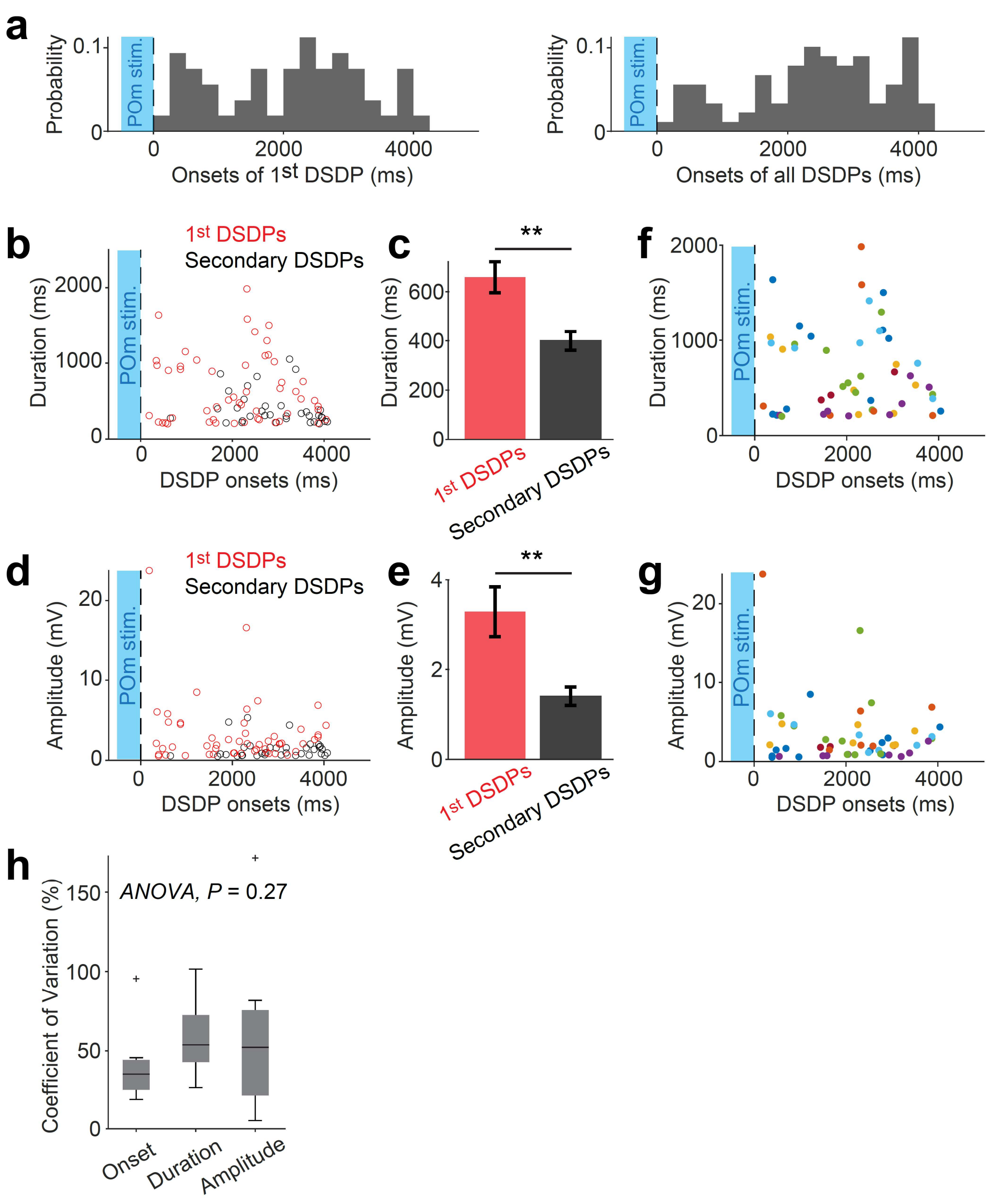

Supplementary Fig. 9 | Temporal Characteristics of DSDP Onsets and Durations Following POm Stimulation (related to Fig. 4).

a Probability distribution of the onset times of the first DSDP following POm stimulation (left histogram) and all DSDPs (right histogram) relative to the start of stimulation. The shaded blue area represents the period of POm stimulation. **b** Scatter plot of DSDP durations as a function of their onset times. Red circles represent the first DSDP, while black circles indicate secondary DSDPs. **c** On average, the duration of the first DSDPs were longer than the secondary DSDPs (1st DSDP : 659 ± 63 ms, n = 52 events; secondary DSDPs : 400 ± 38 ms, n = 34 events, means ± s.e.m; P = 0.0027). **d** Same as **b**, but for the amplitude. **e** On average, the first DSDPs had larger amplitudes than secondary DSDPs (1st DSDP : 3.29 ± 0.55 mV, n = 52 events; secondary DSDPs : 1.40 ± 0.20 mV, n = 34 events, means ± s.e.m; P = 0.009). **f** Scatter plot of first DSDP durations as a function of their onset times for individual cells, where each cell is represented in a different color. **g** Same as **f**, but for the amplitude. **h** Coefficient of

variation within cells for the onset, duration and amplitude of the first DSDP. Source data are provided as a Source Data file.

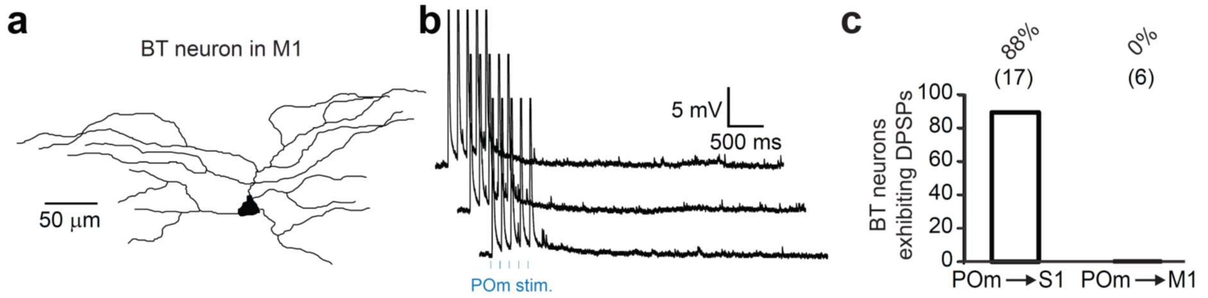

Supplementary Fig. 10 | BT neurons in M1 do not exhibit DSDPs (related to Fig. 4).
a Example of the morphological reconstruction of a BT neuron located in M1. **b** Dendritic recordings of a BT neuron in M1 during and after the photostimulation of POm. Unlike for BT neurons in S1, no DSDPs were observed following the stimulation of POm. **c** Fraction of BT neurons exhibiting DSDPs upon POm stimulation in S1 and in M1. None of the cells recorded in M1 displayed a DSDP (0 out of 6) while 88% of the cells in S1 did (15 out of 17 neurons). Source data are provided as a Source Data file.

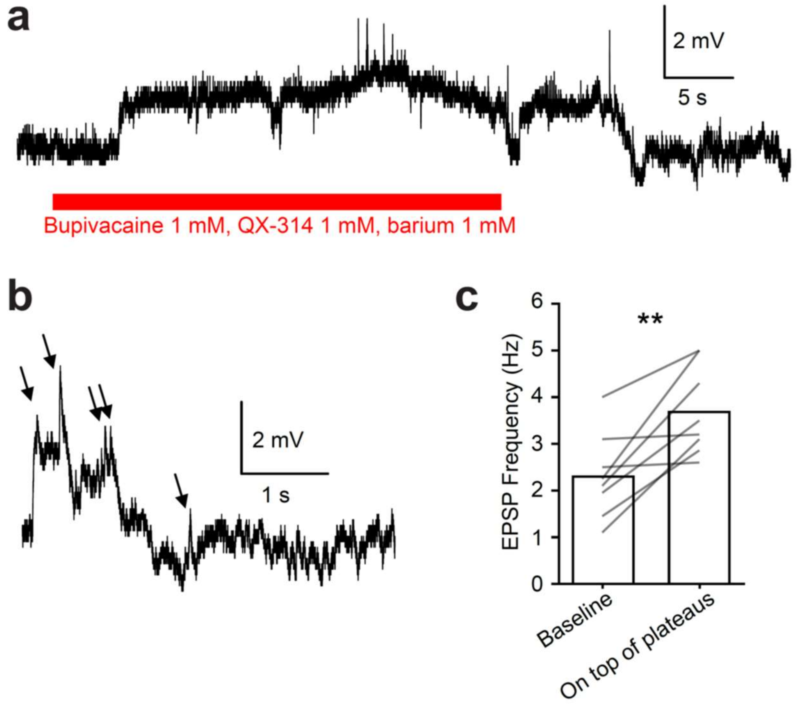

Supplementary Fig. 11 | Effect of K2P blockers on the resting membrane potential (related to Fig. 5).

a Representative dendritic recording showing the effect of a cocktail of K2P channel blockers, including bupivacaine (1 mM), QX-314 (1 mM), and barium (1 mM), as indicated by the red bar. Application of the blockers induced a depolarization of the membrane potential, reflecting a reduction in K2P-mediated resting potassium conductance and an increase in input resistance. The depolarization occurred without external stimulation, highlighting the contribution of K2P channels to maintaining resting membrane potential. Washout of the drug cocktail led to a restoration of the baseline membrane potential, indicating reversibility of the effect. **b** Example trace of a dendritic recording illustrating a DSDP during K2P blockade. Arrows indicate excitatory postsynaptic potentials (EPSPs) occurring on top of the plateau potential. The increase in the numbers of EPSPs suggests enhanced synaptic responsiveness during the plateau state compared to baseline conditions. **c** Quantification of EPSP frequency under baseline conditions and during plateau potentials. The frequency of EPSPs was significantly higher during plateau potentials compared to baseline (in baseline: 2.3 ± 0.32 Hz; on top of plateaus: 3.7 ± 0.35 Hz, means ± s.e.m.; $n = 8$ neurons, $P = 0.004$, paired t-test). This finding highlights a potential role for K2P channel activity in modulating synaptic input integration during altered membrane potential states. Source data are provided as a Source Data file.

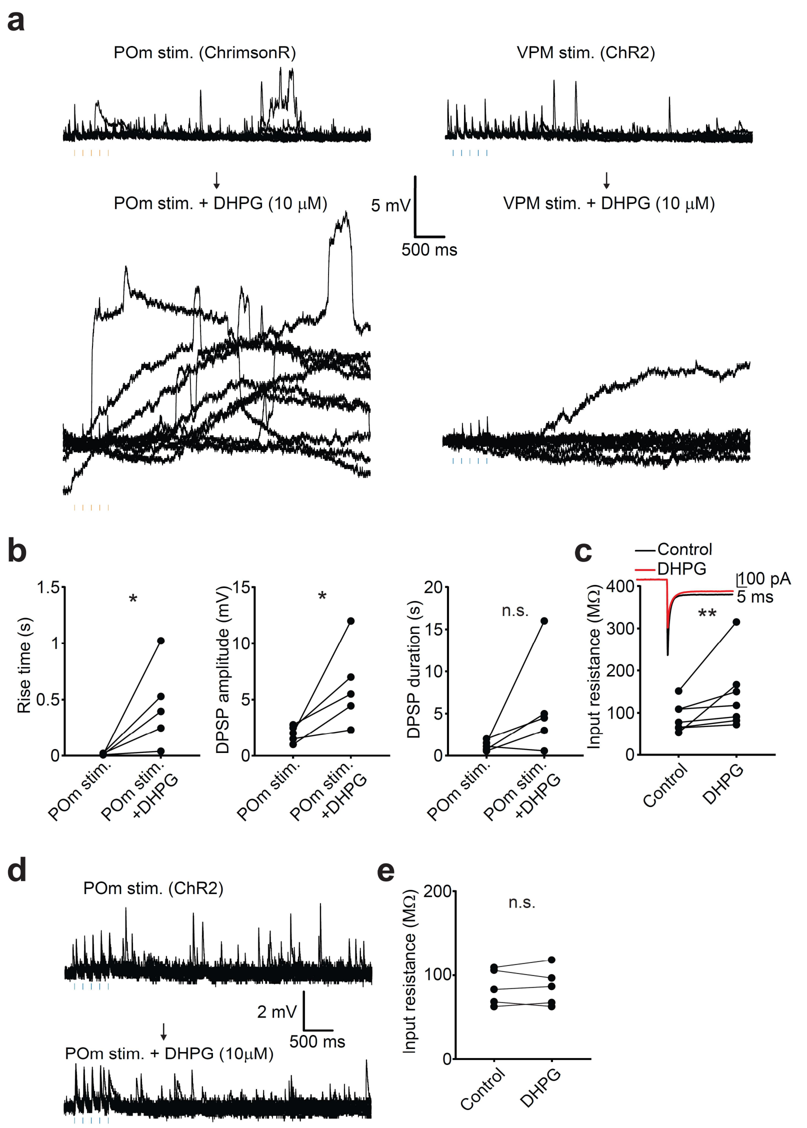

Supplementary Fig. 12 | Effect of DHPG on DSDPs (related to Fig. 6).

a Example of a dendritic recording of a BT cell after photostimulation of ChrimsonR-expressing POm and ChR2-expressing VPM. mGluRI-mediated DSDPs were induced only upon POm stimulation. Subsequent bath perfusion of DHPG (10 µM) increased the number, amplitude, and duration of events after the photostimulation of POm afferent inputs. However, this did not increase the occurrence of DSDPs after VPM stimulation, indicating that DHPG alone is not sufficient to induce them. **b** The rise time of DSDPs, following POm photostimulation, significantly increased in the presence of DHPG (control: 13.7 ± 3.1 ms; DHPG: 446.2 ± 165.5 ms, means ± s.e.m., $n = 5$, $P = 0.05$, paired t-test). It also significantly increased the amplitude of these events (control: 1.9 ± 0.3 mV; DHPG: 6.2 ± 1.6 mV, $n = 5$, $P = 0.05$, paired t-test) but not their duration (control: 1180.9 ± 260.5 ms; DHPG: 5813.3 ± 2660.8 ms, $n = 5$, $P = 0.14$, paired t-test). **c** Perfusion of DHPG significantly increased the dendritic input resistance of BT neurons (control: 79.2 ± 1.2 MΩ; DHPG: 127.2 ± 3.0 MΩ, $n = 8$, $P = 0.0078$, paired t-test). **d** Bath application of DHPG in addition to POm stimulation failed to induce DSDPs in ST neurons. **e** DHPG did not change the input resistance in ST neurons

(control: 85.4 ± 9.4 MΩ; DHPG: 85.6 ± 10.0 MΩ, $n = 5$, $P = 0.88$, paired t-test). Source data are provided as a Source Data file.

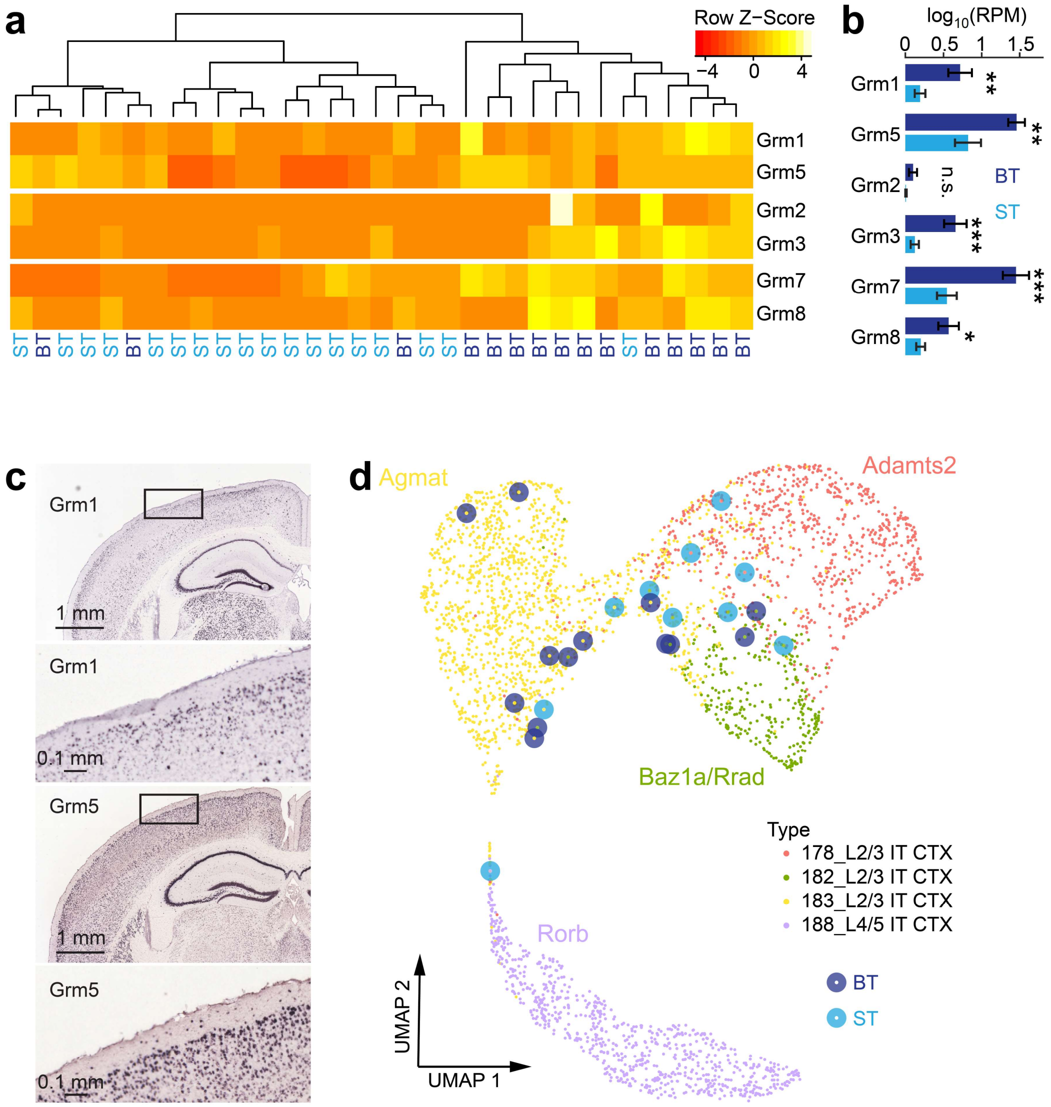

Supplementary Fig. 13 | Multimodal PatchSeq reveals differential molecular profiles as a function of morphological properties in mouse L2/3 neurons (related to Fig. 6).

a Heatmap displaying differential gene expression profiles between BT and ST neurons (expressed as row z-score), highlighting an enrichment of metabotropic glutamate receptor (mGluR) genes (Grm1, Grm2, Grm3, Grm5, Grm7, and Grm8) predominantly in BT neurons ($n = 15$) compared to ST neurons ($n = 18$). Rows correspond to individual mGluR genes, and columns represent neurons, color-coded by subtype. The clustering was generated using unsupervised random forest analysis, revealing distinct molecular signatures associated with BT and ST morphologies. **b** Average expression level, as log10-transformed reads per million (RPM), is significantly higher in BT neurons than in ST neurons for Grm1, 5, 3, 7 and 8 (Grm1: BT 0.72 ± 0.15, ST 0.19 ± 0.07, $P = 0.0027$; Grm5: BT 1.46 ± 0.11, ST 0.82 ± 0.17, $P = 0.0052$; Grm2: BT 0.10 ± 0.05, ST 0.009 ± 0.009, $P = 0.071$; Grm3: BT 0.65 ± 0.15, ST 0.12 ± 0.05, $P = 9.9{\times}10^{-4}$; Grm7: BT 1.45 ± 0.17, ST 0.54 ± 0.13, $P = 1.6{\times}10^{-4}$; Grm8: BT 0.57 ± 0.13, ST 0.20 ± 0.06, $P = 0.013$, t-tests, $n = 15$ BT neurons, $n = 18$ ST neurons). **c** *In situ* hybridization images showing the expression of Grm1 and 5 genes in coronal sections of the mouse brain (mouse

brain ISH data from the Allen Brain Atlas; Grm1 - RP_071218_02_B12 – coronal; Grm5 - RP_050825_01_H07 - coronal). Both Grm1 and 5 expresses at higher levels in the most superficial part of L2/3. **d** Uniform Manifold Approximation and Projection (UMAP) of canonical transcriptomic subtypes of cortical neurons from the somatosensory cortex (from Allen Brain Cell Types RNA-Seq database). PatchSeq data from 23 quality-controlled cells were integrated into this dataset. Classification into BT (bipolar tufted) and ST (simple tufted) subtypes was achieved through post-hoc manual scoring based on detailed morphological reconstructions of labeled cells. The transcriptomic subtypes identified included Agmat, Adamts2, Baz1a/Rrad, and Rorb clusters, as annotated on the UMAP plot. BT neurons (dark blue) show a tendency to map onto the Agmat cluster, whereas ST neurons (light blue) tend to be located within the Adamts2 cluster. This suggests that BT neurons share transcriptional similarities with Agmat-expressing excitatory subtypes whereas ST neurons align more closely with Adamts2-expressing subtypes. Each subtype is color-coded for clarity, and the location of BT (dark blue) and ST (light blue) neurons is highlighted within the UMAP to show their overlap with putative transcriptomic identities. Source data are provided as a Source Data file.

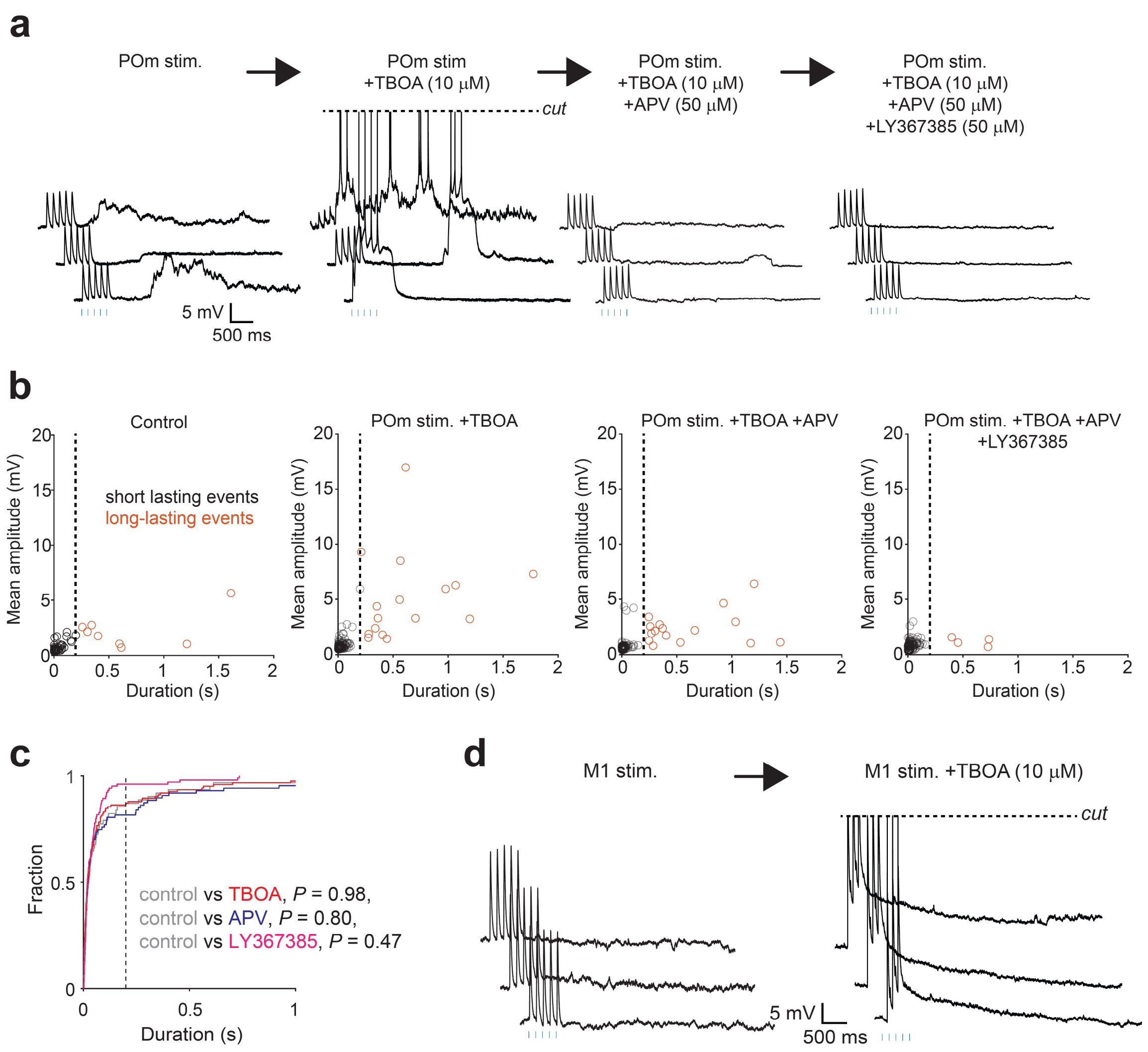

Supplementary Fig. 14 | POm-mediated DSDPs boost NMDA-mediated spiking when the ambient glutamate concentrations are enhanced (related to Fig. 6 and 7).

a Dendritic recordings of a BT neuron in S1 displaying long-lasting and delayed DSDPs after photostimulation of POm afferent inputs. Bath application of 10 µM TBOA, a glutamate reuptake inhibitor that enhances the presence of ambient glutamate, triggering action potentials during DSDPs events. Adding 50 µM APV to the bath prevented the generation of these spikes but DSDPs remained. These were indeed confirmed to be mGluRI-mediated DSDPs as they were abolished when adding 50 µM LY367385 to the bath. **b** Scatter plots showing the duration and mean amplitude of all post-stimulation events automatically detected in BT neurons recordings in S1 (n = 3). Many large and long-lasting events (> 200 ms), corresponding to action potentials or NMDA spikes and DSDPs respectively, were detected under TBOA conditions. The addition of APV reduced the number of large events, but long-lasting DSDPs events remained. Finally, adding LY367385 in the bath resulted in a decrease in the number of detected long-lasting events. **c** Cumulative distribution fractions of all the event durations in control (grey), TBOA (red), TBOA+APV (blue) and TBOA+APV+LY367385 (pink). **d** Dendritic recordings on the same neuron shown in (**a**) after photostimulation of M1 afferents inputs did not produce any DSDPs. M1 photostimulation during bath perfusion of TBOA generated spikes but no delayed spiking events were observed. Source data are provided as a Source Data file.

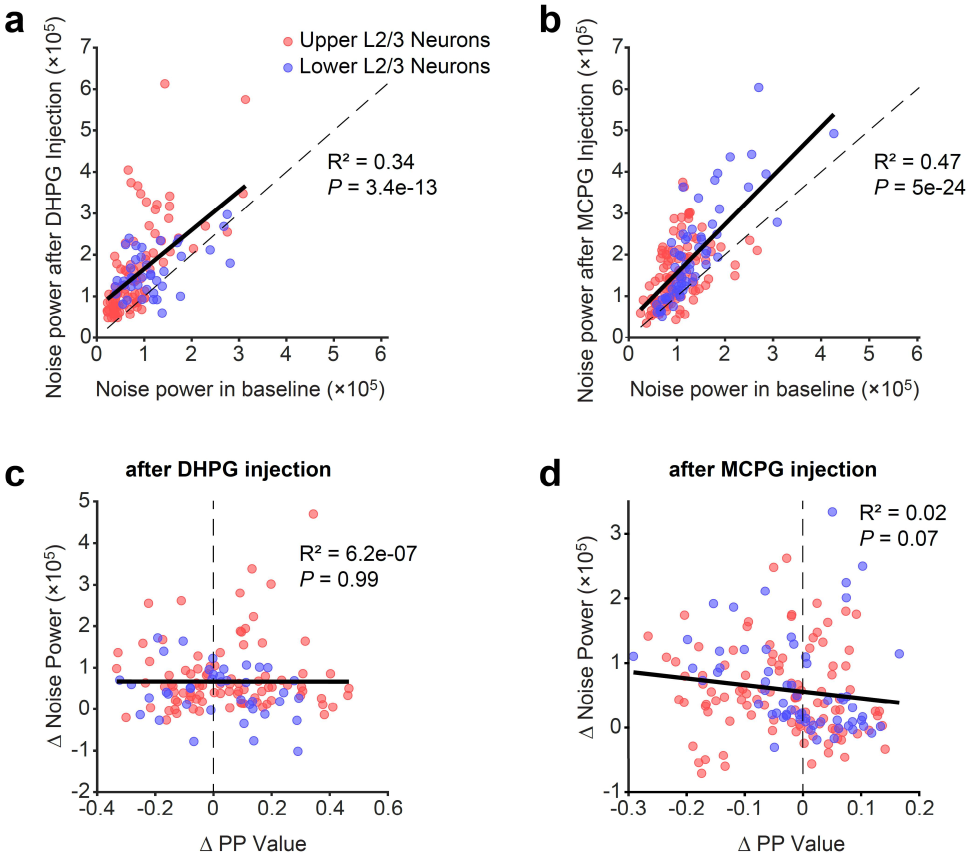

Supplementary Fig. 15 | The prediction power measurements are not sensitive to noise levels in the calcium activity traces (related to Fig. 7).

a Comparison of the noise power (see Methods) between baseline and upon cortical DHPG injections. This indicates that the noise level during calcium imaging slightly increased after DHPG injection. **b** Same comparison after MCPG injection. **c** Comparison of the change in prediction power (PP) (after DHPG injection - baseline) to the change in noise power. No correlation was observed between the change in noise level of the measurement and the change in PP value. **d** Same analysis for the MCPG condition. The noise power was calculated as follows: the calcium activity signal was mean centered to remove any DC offset. It was then transformed into the frequency domain using the Fast Fourier Transform (FFT), and the Power Spectral Density (PSD) was computed. Source data are provided as a Source Data file.

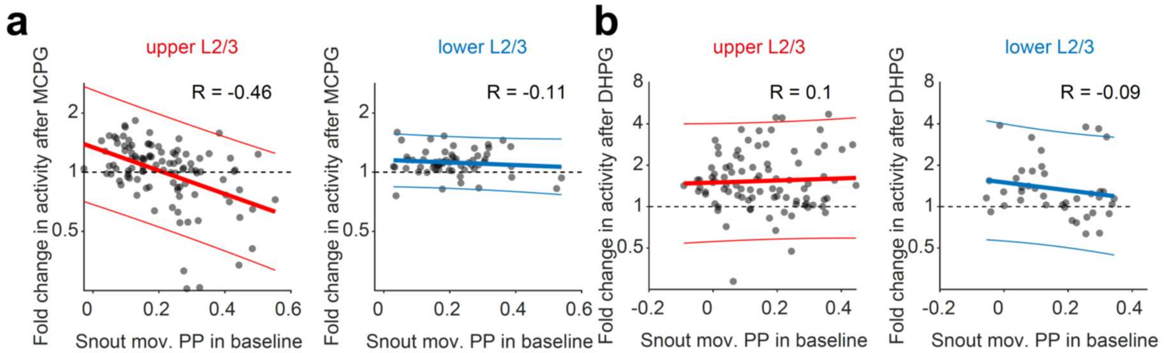

Supplementary Fig. 16 | Relation between movement prediction in baseline and mGluRI modulation of activity (related to Fig. 7).

a Correlation between the snout movement PP in baseline and the change in activity after MCPG injection. Upper L2/3 neurons that show the strongest reduction in activity in MCPG predicted best the snout movement during the baseline recording (n = 104 upper and 61 lower L2/3 neurons, from 3 mice; Pearson's correlation R = -0.46). Lower L2/3 neurons show little to no correlation (R = -0.11). **b** Same for DHPG dataset, little to no correlation was observed for the upper and lower L2/3 neurons (n = 90 upper and 40 lower L2/3 neurons, from 5 mice; R = 0.1 and -0.09 respectively). Source data are provided as a Source Data file.

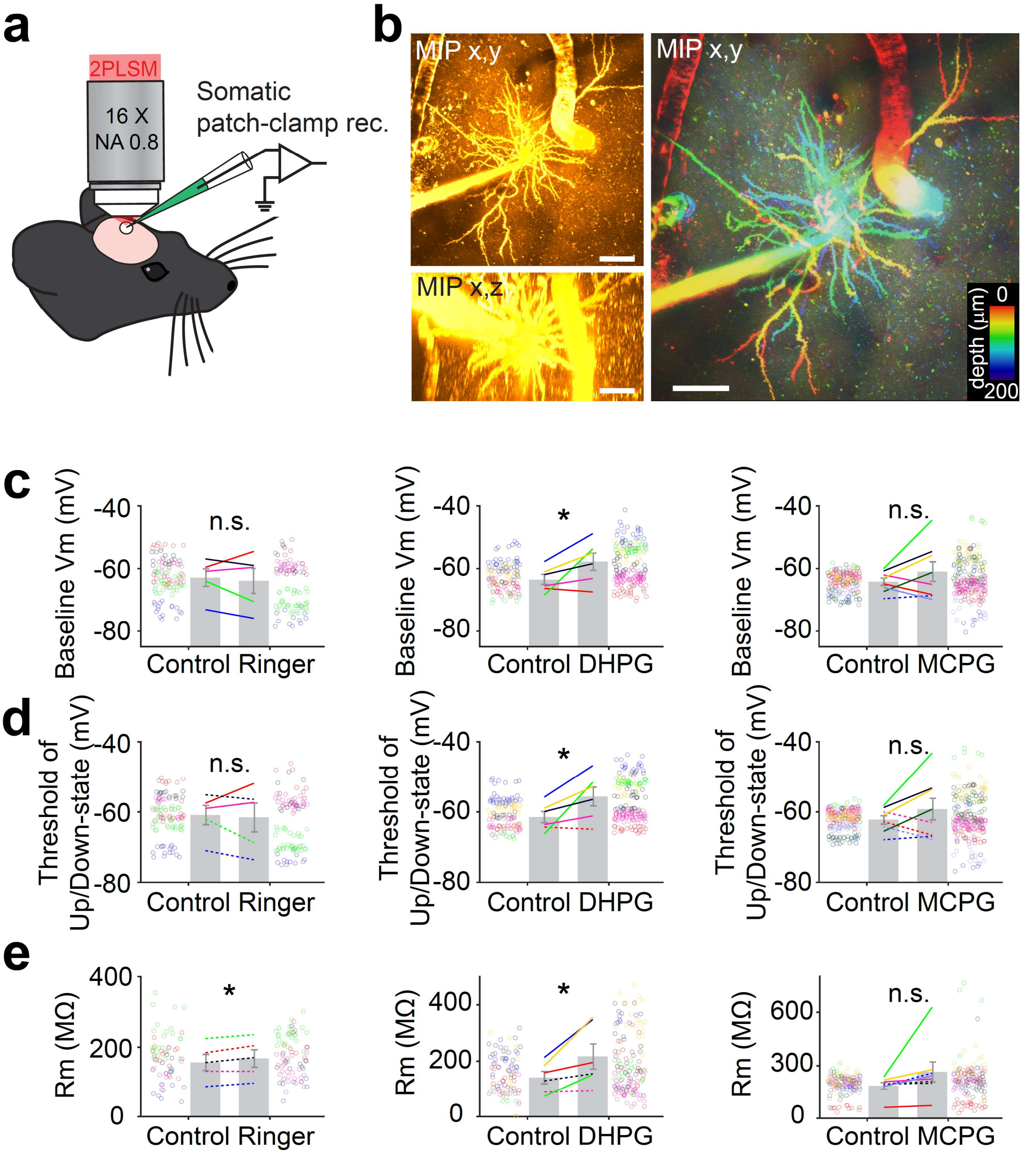

Supplementary Fig. 17 | In vivo whole-cell recordings from superficial layer 2/3 pyramidal neurons reveal increased intrinsic excitability following mGluRI activation during whisker stimulation (related to Fig. 7).

a Schematic of the experimental setup. Whole-cell somatic patch-clamp recordings were performed on superficial L2/3 PNs in S1 of anesthetized mice. Patched neurons were filled with Alexa Fluor-488 via the patch pipette to enable morphological characterization using 2PLSM. **b** Example of maximum intensity projections of the Alexa Fluor signal (top view, MIP x,y and side view, MIP x,z) from a patched neuron obtained from a 2PLSM z-stack. On the right side, MIP x,y view color-coded by imaging depth confirming the characteristic morphology of a BT neuron. Scale bars: 50 µm. **c** Changes in baseline membrane potential following topical application of Ringer solution, DHPG (5 mM), or MCPG (0.5 or 1 mM). DHPG significantly depolarized the membrane potential (n = 6 neurons; P = 0.05, paired t-test),

whereas Ringer's solution and MCPG had no significant effect (Ringer: n = 5 neurons, P = 0.63; MCPG: n = 8 neurons, P = 0.22). **d** Changes in the threshold of Up and Down states following drug application. DHPG significantly increased the threshold (n = 6 neurons, P = 0.05, paired t-test), whereas Ringer (n = 5, P = 0.77) and MCPG (n = 8, P = 0.23) had no significant effect. **e** Effect of Ringer, DHPG, and MCPG on input resistance. Ringer solution significantly increased input resistance (n = 5 neurons, P = 0.03, paired t-test), but individually, none of neurons exhibited a significant increase ($P > 0.05$, two-sample t-test). DHPG also significantly increased input resistance (n = 6 neurons, P = 0.04, paired t-test), while MCPG had no effect (n = 8 neurons, P = 0.13). For (**c–e**), circles represent individual trial measurements, with colors indicating different cells. Dashed lines denote the average for neurons that did not exhibit a significant change (two-sample t-test), while continuous lines indicate those neurons with significant changes. Averages of individual neurons were tested with paired t-tests and bars and error bars represent their mean ± s.e.m. across cells. Overall, mGluRI activation with DHPG increased the excitability of BT neurons. Blocking mGluRI signaling with MCPG was variable and did not, on average, reduce excitability. This lack of effect may be due to a low basal level of mGluRI activation under control conditions. Since the recordings were performed under anesthesia, it is possible that the mGluRI signaling is strongly diminished and cannot be further reduced with MCPG. Furthermore, the effects of DHPG and the absence of MCPG effects align well with the conclusions drawn from the *in vivo* calcium imaging experiments (Fig. 7) where the effect on single neurons was variable, and the overall activity of neurons did not significantly decrease upon MCPG. Source data are provided as a Source Data file.
