# Supplementary material for: Thalamocortical feedback selectively controls pyramidal neuron excitability
Source: Nat Commun. 2025 Jul 1;16:5663. doi: 10.1038/s41467-025-60835-w (PMC12215560; doi:10.1038/s41467-025-60835-w)
Supplement: Supplementary file 2 — Reporting Summary [file 41467_2025_60835_MOESM2_ESM.pdf]

nature portfolio

Corresponding author(s): Anthony Holtmaat

Last updated by author(s): Jan 10, 2024

Reporting Summary

Nature Portfolio wishes to improve the reproducibility of the work that we publish. This form provides structure for consistency and transparency in reporting. For further information on Nature Portfolio policies, see our Editorial Policies and the Editorial Policy Checklist.

Statistics

For all statistical analyses, confirm that the following items are present in the figure legend, table legend, main text, or Methods section.

n/a	Confirmed	
☐	☒	The exact sample size (*n*) for each experimental group/condition, given as a discrete number and unit of measurement
☐	☒	A statement on whether measurements were taken from distinct samples or whether the same sample was measured repeatedly
☐	☒	The statistical test(s) used AND whether they are one- or two-sided<br>*Only common tests should be described solely by name; describe more complex techniques in the Methods section.*
☒	☐	A description of all covariates tested
☐	☒	A description of any assumptions or corrections, such as tests of normality and adjustment for multiple comparisons
☐	☒	A full description of the statistical parameters including central tendency (e.g. means) or other basic estimates (e.g. regression coefficient) AND variation (e.g. standard deviation) or associated estimates of uncertainty (e.g. confidence intervals)
☐	☒	For null hypothesis testing, the test statistic (e.g. *F*, *t*, *r*) with confidence intervals, effect sizes, degrees of freedom and *P* value noted<br>*Give P values as exact values whenever suitable.*
☒	☐	For Bayesian analysis, information on the choice of priors and Markov chain Monte Carlo settings
☒	☐	For hierarchical and complex designs, identification of the appropriate level for tests and full reporting of outcomes
☐	☒	Estimates of effect sizes (e.g. Cohen's *d*, Pearson's *r*), indicating how they were calculated

Our web collection on statistics for biologists contains articles on many of the points above.

Software and code

Policy information about availability of computer code

Data collection	Scanimage 2016b (https://www.mbfbioscience.com/products/scanimage/) was used to image neuronal activity (http://www.scanimage.org) running on MATLAB 2017a.<br>pClamp 10.5 (Molecular Devices) was used for acquisition of electrophysiological data.<br>Neurolucida (MBF Bioscience) was used to reconstruct neurons morphology.
Data analysis	Custom-written MATLAB scripts (Matlab 2018a and 2020b; MATHWORKS) and custom-written Fiji (https://imagej.net/Fiji) plugins were used to process the 2-photon images and custom-written scripts were used to analyse the behavior and calcium imaging data.<br>Clampfit 10 (Molecular Devices), Origin 2021 (OriginLab Corporation), and Prism (GraphPad) were used for analysis of electrophysiological data.<br>Python and Deeplabcut v2.2.0.2 (https://github.com/DeepLabCut/DeepLabCut) were used to extract animal movements from video recordings.<br>R v4.0.3 was used to build the custom-written Random Forests model used in this study.

For manuscripts utilizing custom algorithms or software that are central to the research but not yet described in published literature, software must be made available to editors and reviewers. We strongly encourage code deposition in a community repository (e.g. GitHub). See the Nature Portfolio guidelines for submitting code & software for further information.

Data

Policy information about availability of data

All manuscripts must include a data availability statement. This statement should provide the following information, where applicable:

- Accession codes, unique identifiers, or web links for publicly available datasets
- A description of any restrictions on data availability
- For clinical datasets or third party data, please ensure that the statement adheres to our policy

We have provided a full data availability statement in the manuscript.

Research involving human participants, their data, or biological material

Policy information about studies with human participants or human data. See also policy information about sex, gender (identity/presentation), and sexual orientation and race, ethnicity and racism.

Reporting on sex and gender	N/A
Reporting on race, ethnicity, or other socially relevant groupings	N/A
Population characteristics	N/A
Recruitment	N/A
Ethics oversight	N/A

Note that full information on the approval of the study protocol must also be provided in the manuscript.

Field-specific reporting

Please select the one below that is the best fit for your research. If you are not sure, read the appropriate sections before making your selection.

☒ Life sciences ☐ Behavioural & social sciences ☐ Ecological, evolutionary & environmental sciences

For a reference copy of the document with all sections, see nature.com/documents/nr-reporting-summary-flat.pdf

Life sciences study design

All studies must disclose on these points even when the disclosure is negative.

Sample size	We did not employ statistical methods to pre-determine sample sizes; however, our sample sizes align with those documented in prior publications (refer to Bureau et al.[3], Sermet et al.[6], Williams et al.[26], Lavzin et al.[38]).
Data exclusions	Electrophysiological recordings were excluded if they did not meet standard inclusion criteria such as access resistance. No data sets were excluded from the analysis if recordings were complete. In the case of in vivo cellular imaging data, exclusion criteria were applied if motion artifacts could not be post-hoc rectified or if the field of view could not be retrieved after the drug injection.
Replication	All results were replicated at least in 3 mice for electrophysiological and in vivo calcium imaging data and at least 2 mice for the anterograde labelling analysis.
Randomization	No randomization was used in our experiments as no selection bias was introduced.
Blinding	For the anterograde labelling analysis, experimenters were blinded to the condition (Extended Data Fig. 4).<br>For the rest of the study, investigators were not blinded to the experimental conditions. Analysis of the electrophysiological data was semi-automated and therefore unbiased except for Extended Data Fig. 7 were the types of postsynaptic events were manually selected to highlight their differences. Analysis of the calcium imaging data was performed semi-automated.

Reporting for specific materials, systems and methods

We require information from authors about some types of materials, experimental systems and methods used in many studies. Here, indicate whether each material, system or method listed is relevant to your study. If you are not sure if a list item applies to your research, read the appropriate section before selecting a response.

Materials & experimental systems

n/a	Involved in the study
☒	☐ Antibodies
☒	☐ Eukaryotic cell lines
☒	☐ Palaeontology and archaeology
☐	☒ Animals and other organisms
☒	☐ Clinical data
☒	☐ Dual use research of concern
☒	☐ Plants

Methods

n/a	Involved in the study
☒	☐ ChIP-seq
☒	☐ Flow cytometry
☒	☐ MRI-based neuroimaging

Animals and other research organisms

Policy information about studies involving animals; ARRIVE guidelines recommended for reporting animal research, and Sex and Gender in Research

Laboratory animals	Mouse/C57BL/6JRj (Janvier Labs; https://janvier-labs.com/fiche_produit/2-c57bl-6jrj/)<br>Mouse/ Pvalbtm1(cre)Arbr/J (PVcre) (The Jackson Laboratory; MGI:3590684 https://www.jax.org/strain/008069, RRID: IMSR_JAX:008069).<br>8-12 weeks old mice were used for all the experiments.
Wild animals	This study did not involve the use of wild animals.
Reporting on sex	Males and females were used.
Field-collected samples	No field-collected samples were used in this study.
Ethics oversight	All procedures were conducted in accordance with the guidelines of the Federal Food Safety and Veterinary Office of Switzerland and in agreement with the veterinary office of the Canton of Geneva (license numbers GE12219B, GE/74/18 and GE253A).

Note that full information on the approval of the study protocol must also be provided in the manuscript.

Plants

Seed stocks	N/A
Novel plant genotypes	N/A
Authentication	N/A
